# Light intensity defines growth and photopigment content of a mixed culture of purple phototrophic bacteria

**DOI:** 10.3389/fmicb.2022.1014695

**Published:** 2022-10-19

**Authors:** Marta Cerruti, Jeong-Hoon Kim, Martin Pabst, Mark C. M. Van Loosdrecht, David G. Weissbrodt

**Affiliations:** Department of Biotechnology, Delft University of Technology, Delft, Netherlands

**Keywords:** anaerobic wastewater treatment, water resource recovery, PNSB, irradiance effects, pigments

## Abstract

Purple bacteria (PPB), anoxygenic photoorganoheterotrophic organisms with a hyper-versatile metabolism and high biomass yields over substrate, are promising candidates for the recovery of nutrient resources from wastewater. Infrared light is a pivotal parameter to control and design PPB-based resource recovery. However, the effects of light intensities on the physiology and selection of PPB in mixed cultures have not been studied to date. Here, we examined the effect of infrared irradiance on PPB physiology, enrichment, and growth over a large range of irradiance (0 to 350 W m^−2^) in an anaerobic mixed-culture sequencing batch photobioreactor. We developed an empirical mathematical model that suggests higher PPB growth rates as response to higher irradiance. Moreover, PPB adapted to light intensity by modulating the abundances of their phototrophic complexes. The obtained results provide an in-depth phylogenetic and metabolic insight the impact of irradiance on PPB. Our findings deliver the fundamental information for guiding the design of light-driven, anaerobic mixed-culture PPB processes for wastewater treatment and bioproduct valorization.

## Introduction

Biological wastewater treatment is an established technique to treat polluted waters before discharge in the environment or re-use. Several microbial processes have been harnessed to achieve biological nutrient removal (BNR) and recovery of resources embedded in the wastewater, e.g., conventional activated sludge, biofilm systems, granular sludge, anaerobic processes, and phototrophic systems (Pell and Wörman, 2006). Within the latest incentives to reach circular economy goals, environmental biotechnologies are developed to capture nutrients and valorize the microbial biomass (Guest et al., 2009; Kehrein et al., 2020).

Purple phototrophic bacteria (PPB) attract an increasing interest for wastewater treatment combined with the capture of nutrients into a valuable biomass (Alloul et al., 2018; Hülsen et al., 2018; Puyol et al., 2019). These anoxygenic photoorganoheterotrophs can use numerous organic and inorganic substrates as carbon and/or electron sources, growing with biomass yields over substrate close to unity by thriving on infrared light energy (Capson-Tojo et al., 2020; Cerruti et al., 2020a). PPB biomasses can be valorized for biogas and bioplastic productions, as fertilizer, and feed additive. Central to their phototrophic metabolism, PPB synthetize photopigments to capture the light and produce cellular energy (Yurkov and Beatty, 1998). Bacteriochlorophylls and carotenoids, placed in the intracellular membranes, harvest light. Bacteriochlorophylls present high quantum yields: almost all the energy from the photons is transferred and used for chemical energy production (Wraight and Clayton, 1974). Carotenoids are accessory pigments that transfer light to the reaction centers and protect the cells from photooxidation damage (Cerullo et al., 2003). Both types of photopigments can find industrial applications. Bacteriochlorophylls can for instance be used as biomarkers in drug development (Pantiushenko et al., 2015). Carotenoids of natural and artificial synthesis are used as food additives and food dyes (Cardoso et al., 2017).

Artificial light supply accounts for one of the major costs in photobiosystems (Blanken et al., 2013), and therefore its effects on the microbial bioprocess need to be carefully estimated. In their growth physiology, phototrophic organisms can face two main regimes driven by light intensity, namely: light limitation (growth linked to light intensity), and light saturation (Carvalho et al., 2011). PPB responses to light variations have been extensively studied in pure cultures and membrane extracts (Kuo et al., 2012; Imam et al., 2015). They adapt the number and disposition of the light-harvesting complexes to the environmental conditions (Jackson et al., 2012; Muzziotti et al., 2017). The growth rates of PPB are as well dependent on light intensity (Katsuda et al., 2004), increasing with irradiance up to a saturation threshold (325 μmol photons s^−1^ m^−2^ for purple sulfur bacteria and at around 60 μmol photons s^−1^ m^−2^ for purple non-sulfur bacteria; Ritchie, 2013; Ritchie and Mekjinda, 2015).

Light is attenuated by the biomass and by the specific photopigment content (Lee et al., 2015). Shading effects have a substantial impact on the light available for the cells to grow, factually decreasing it. These effects are often neglected in PPB studies, where only the incident light is measured. An efficient growth of PPB in mixed culture and nutrient removal was achieved at low light intensities (1.4 W m^−2^, or 11 μmol photons s^−1^ m^−2^ of IR light; Dalaei et al., 2020), but the minimum quantum requirement (the minimal irradiance necessary for growth) and the ‘affinity constant for light’, are not reported for PPB. Despite the pivotal importance of light for PPB growth, little is known about the structure, robustness, and functionality of PPB microbial communities under light variations.

Here, we evaluated the effect of 9 different light intensities from 350 to 0 W m^−2^ on the PPB community structure and growth kinetics, along with their photopigment content in a mixed culture. Using this wide range of light intensities, we evaluated the growth limits of a PPB enrichment, linked to photopigment content and enzymatic functionality. The combination of quantitative mixed-culture biotechnology, physiological and pigment measurements, and metaproteomic analysis was efficient to understand the impacts on the metabolic regulation of PPB mixed cultures in function of infrared light irradiances. The knowledge developed on the adaptation strategies of a PPB community to variations in photonic energy supply provides the fundamental knowledge necessary to develop PPB-based technologies for water resource recovery.

## Materials and methods

### Reactor setup and operational conditions

A 2.5-L, stirred-tank, sequencing batch photobioreactor with a 11-cm diameter and 2.0-L working volume connected to a controller system (In-Control and Power units, Applikon, Netherlands) was used to cultivate the PPB enrichment on an acetate-based synthetic wastewater. The photobioreactor consisted of a cylindrically shaped vessel with a curved bottom made of borosilicate glass [transmittance ≥90% in the wavelength range of the filtered light (Xia et al., 2017)]. The temperature was controlled at 30° C through a finger-type heat exchanger connected to a thermostat (WK 500, Lauda, Germany). Argon gas (99% purity, Linde, NL) was continuously sparged in the reactor (excluding the settling phase) to ensure anaerobic conditions. Uniform mixing was provided with a custom-made anchor stirrer. Silicon blades were attached to the anchor stirrer to wipe the inner surface of the reactor and minimize biofilm growth on the reactor surface which can severely interfere the penetration of light into the reactor. Residual biofilm growth on probes and stirrer was removed once a week.

The system was operated in a continuously-illuminated sequencing batch reactor (SBR) mode with an 8-h cycle composed of 5 min of feed, 281 min of reaction, 4 min of purging of mixed liquor, 210 min of settling, 5 min of discharge of the supernatant, and 5 min of idle. The purge volume of mixed liquor was adjusted based on the conditions to maintain an SRT of 31 h. The volume exchange ratio was set to 50% of the working volume.

The cultures were supplied with a synthetic wastewater medium composed of (per L): 0.914 g C_2_H_3_O_2_·3H_2_O, 0.14 g KH_2_PO_4_, 0.21 g K_2_HPO_4_, 1 g NH_4_Cl, 2 g MgSO_4_·7H_2_O, 1 g CaCl_2_·2H_2_O, 1 g NaCl, as well as 2 ml of trace elements and 2 ml of vitamins solutions. The stock solution of vitamins was composed of (per L): 200 mg thiamine–HCl, 500 mg niacin, 300 mg ρ-amino-benzoic acid, 100 mg pyridoxine–HCl, 50 mg biotin, and 50 mg vitamin B12. The trace elements solution was made of (per L): 1100 mg Na_2_EDTA·2 H_2_O, 2000 mg FeCl_3_·6 H_2_O, 100 mg ZnCl_2_, 64 mg MnSO_4_·H_2_O, 100 mg H_3_BO_3_, 100 mg CoCl_2_·6 H_2_O, 24 mg Na_2_MoO_4_·2 H_2_O, 16 mg CuSO_4_·5 H_2_O, 10 mg NiCl_2_·6 H_2_O, and 5 mg NaSeO_3_. The cultivation medium was buffered at pH 7.0 with 4 g L^−1^ 4-(2-hydroxyethyl)-1-piperazineethanesulfonic acid (HEPES).

The SBR was started at 0.7 mg volatile suspended solids (VSS) L^−1^ with an in-house mother mixed culture of PPB maintained under non-limiting IR light at 350 W m^−2^ under SBR conditions as in Cerruti et al., (2020b). Briefly, each SBR cycle was composed of a reaction phase (4.75 h), a settling phase (3 h), and discharge, feed, and idle phases (5 min). The experiments at high irradiances (350, 264, 175, and 87 W m^−2^) and the experiments at low irradiances (30, 15, 7, 3, and 0 W m^−2^) were subsequently performed in two different periods over the year. Between these two sets of experiments, the SBR was maintained at 350 W m^−2^ under SBR conditions as well, for a period of 4 months. Due to unknown variations in the operations, during the first period, the genus *Thiobaca* was dominant, while the genus *Rhodopseudomonas* was predominant at the beginning of the second one.

### Infrared light irradiance and measurements

The photobioreactor was placed in a dark hood to prevent external light penetration. Light was provided from two opposite sides of the reactor with halogen lamps (Philips Plusline ES 120 W R7S 230 V, Philips, NL), filtered through two Black 962 Infrared Transmitting Perspex Acrylic Sheets (Black Perspex 962, Plastic stocktist, United Kingdom) for supplying infrared (IR) wavelengths (λ) > 700 nm to promote PPB growth. A dimmer was used to tune the light intensity to nominal set points (GAMMA, NL). Incident IR light intensity at the surface of the reactor was measured with a pyranometer (CMP3; Kipp & Zonen, NL). To evaluate the effects of different light intensities, the biomass was subjected to 9 different incident light conditions: 350, 264, 175, 87, 30, 15, 7, 3, and 0 W m^−2^. To convert the measured light intensity (W m^−2^) to a photon flux (μmol photons s^−1^ m^−2^), the average of the photon flux per wavelength was used, calculated according to Equation (1):


(1) $$\Phi =\frac{I\cdot \lambda \cdot 10^{6}}{N\cdot h\cdot c}=\frac{\mu molphotons}{m^{2}\cdot s}$$

with Φ the photon flux (μmol photons s^−1^ m^−2^), *I* the irradiance (W m^−2^, i.e., J s^−1^ m^−2^), λ the wavelength (m), *N* the Avogadro number (6.02·10^23^ photons mole^−1^), *h* the Planck’s constant (6.63·10^−34^ J s), and *c* the speed of light (2.88·10^8^m s^−1^).

### Specific light supply rate

The specific light supply rate (rEX) defines the amount of light available per gram of biomass. It was calculated as in Sforza et al. (2015):


(2) $$rEX=\frac{PDF\cdot A}{X_{av\;\;\cdot }\;V}$$

with *PFD* the photon flux (μmol photons m^−2^ s^−1^), *A* the irradiated surface (calculated from the reactor geometry; m^2^), *X_av_* the average biomass concentration in the cycles for each condition (g L^−1^), *V* the reactor working volume (L).

### Sample collection

Each light condition was maintained for 14 days (10.8 STRs). Mixed liquor samples for community composition analysis, photopigment concentration, and acetate removal were collected from day 10–11 to day 14 after setting a new light intensity condition, i.e., corresponding to 7.7 to 10.8 SRTs from the start of the new irradiation regime.

### Measurement of biomass concentration

The biomass concentrations in the mixed liquor obtained at the different IR-light irradiances were measured by spectrophotometry (Biochrom, Libra S11, US) through absorbance at 660 nm (Abs_660_). A calibration curve was established to correlate Abs_660_ to the biomass concentration (g VSS): c = 1.4 Abs_660_. Volatile suspended solids (VSS) were measured following the Standard Methods (Water Environment Federation, 1999).

### Analysis of bacterial community compositions by amplicon sequencing

The compositions of the bacterial communities present in the mixed liquors under the different IR light irradiances were characterized from the biomass samples by V3-V4 16S rRNA gene-based amplicon sequencing as detailed in Cerruti et al. (2020b). The DNA extracts were sent to Novogene (United Kingdom) for library preparation and amplicon sequencing. The fastq files received from Novogene were analyzed with the QIIME2 pipeline (Bolyen et al., 2019). The detailed sequencing result are available in the NCBI database under the Bioproject ID PRJNA799236.

### Extraction and analysis of photopigments

Bacteriochlorophyll and carotenoid photopigments were extracted from the PPB biomasses. Their mass fractions were quantified and compared at the different IR light irradiances.

#### Photopigments extraction

Prior to pigments extraction, biomass was freeze-dried for 12 h at −50°C and at a pressure of 0.05 mbar, to remove water from the pellets and facilitate polar solvent penetration.

Hexane/methanol/water in proportions of 2:1:1 (v:v:v) were used to extract the photopigments. Hexane and methanol were mixed and pre-chilled at 4°C overnight. A volume of 1,200 μl of this solution was added to the biomass in a cold and dark environment. The cells were vortexed with intermittent cooling on ice to promote the extraction of non-polar compounds. Once completely resuspended, the cells were incubated on ice for 5 min. Water was added to promote the separation of the photopigments (non-polar) from cell debris, and the samples were centrifuged for 5 min at 17000 x g, resulting in two separate phases. The upper phase, non-polar, consisting of methanol and hexane, was collected for spectrophotometric quantification of the photopigments.

#### Quantification of photopigments

The concentration of the photopigments in the samples was calculated according to the Lambert–Beer equation:


(3) $$A=\varepsilon_{\lambda }\cdot C_{p}\cdot D$$

Where *A* is the measured absorbance at the peak wavelength (lycopene = 473 nm, bchl = 776 nm), *ε_λ_* the molar absorption coefficient (lycopene = 1.72*10^5^ M^−1^ g_photopigment_^−1^, bchl = 6*10^4^ M^−1^ g_photopigment_^−1^ (Bóna-Lovász et al., 2013)) of the compound at specific wavelength *λ*, *Cp* the molar concentration of the photopigment, and *D* the pathlength of light in the spectrophotometric cuvette (0.5 cm).

To calculate the photopigment mass fraction, the photopigments molar concentration was divided by the bacterial mass before the extraction and multiplied by the final volume of solvent (hexane) used for the elution.

### Calculation of growth kinetic and stoichiometric parameters

Growth rate.

Biomass-specific growth rates μ (h^−1^) were calculated from the mixed culture as:


(4) $$\mu =\ln\left(\frac{X_{1}}{X_{0}}\right)\cdot \frac{1}{T_{1}-T_{0}}$$

with *X_0_* and *X_1_* the biomass concentration at the beginning and end of the SBR growth phase, respectively, and *T_0_* and *T_1_* the time at the beginning and end of the SBR growth phase, respectively.

The growth rates were fitted to the light intensity with a generic logistic function (Equation 5) through the lsqcurvefit function of MATLAB (R2018b, MathWorks, Natick, MA^1^). It is a nonlinear least-square solver that finds the coefficients that best fit a given nonlinear function.


(5) $$y=\frac{a}{1+e^{\left[-b\left(x-c\right)\right]}}$$

### Metaproteomic analysis

High-resolution analysis of the metaproteome of the mixed culture was performed on selected biomass samples collected at the end of the experimental periods at incident irradiances of 350, 87 and 15 W m^−2^ to evaluate the differences in pathway expression. After cell lysis and protein extraction, proteins were digested with trypsin and purified by solid phase extraction. The resulting peptides were analyzed by a one-dimensional shotgun metaproteomic approach using a QE plus Orbitrap mass spectrometer (Kleikamp et al., 2022). The datasets were analyzed using a taxonomy-focused UniprotKB database. The *de novo* identification of the taxonomies has been described in more detail by Kleikamp et al. (2021). The list of identified proteins with their accession number is available in Supplementary material 1.

The area of the peak for each protein was normalized for the total area of each sample, and expressed as its percentage. A statistical analysis was carried on the samples triplicates using Rstudio (RStudio Team, 2020). The 87 W m^−2^ condition was used as a reference point. The protein average and the foldchange were calculated. A *t*-test was used to evaluate the significance in protein expression difference between the three conditions. Proteins with a value of p below 0.05 were marked as significantly differently expressed, comparing 350 W m^−2^ vs 87 W m^−2^ conditions and 15 W m^−2^ vs 87 W m^−2^ conditions. Proteins with a value of *p* <0.05 and a log_2_-fold-change >0.5 or < −0.5 were considered over- or underexpressed compared to the reference set (87 W m^−2^), respectively.

The UniProt accession identifiers were translated to KEGG IDs through BlastKOALA (Kanehisa et al., 2016) server. Protein classification was done according to the KEGG database. A manual classification of the entries not represented in the KEGG database was further performed.

## Results

### Biomass growth and acetate consumption

**Figure 1:**
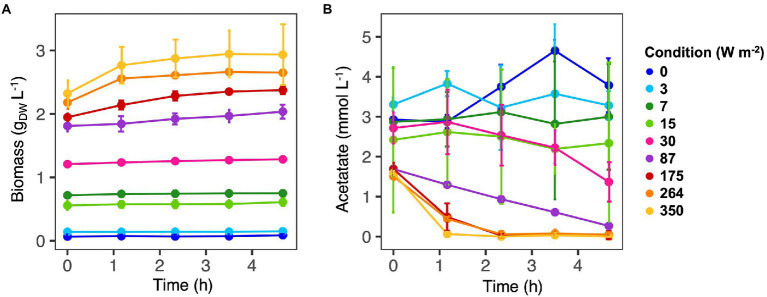
Biomass **(A)** and acetate **(B)** concentrations along the SBR cycles. Values are expressed as the average value for each time point. Samples were collected for three cycles. At high light intensities (350–87 W m^−2^) acetate was fully consumed at the end of the reaction cycles. At low light intensity (30–0 W m^−2^) acetate was still present at the end of the reaction phase and biomass growth was negligible.

Incident IR light intensities supplied at the surface of the anaerobic sequencing batch photobioreactor impacted biomass growth and nutrient capture by the PPB mixed culture. Under high light intensities of 350 and 264 W m^−2^, the biomass reached a concentration plateau of 2.8 ± 0.2 g_VSS_ L^−1^ (Table 1) within the first 70 min of cycle operation (Figure 1A). At medium light intensities of 175 and 87 W m^−2^, the biomass grew to lower concentrations and continuously during the 4 h of cycle operation. At lower light intensities of 30–0 W m^−2^, the biomass growth was almost negligible (Figure 1A).

**Table 1:** Biomass and acetate concentration at the end of the reaction phase and specific growth rates calculated for each condition.

	IR light irradiance conditions (W m^−2^)								
	0	3	7	15	30	87	175	264	350
Biomass* (g_VSS_ L^−1^)	0.09 ± 0.02	0.15 ± 0.01	0.75 ± 0.05	0.61 ± 0.06	1.28 ± 0.02	2.04 ± 0.11	2.37 ± 0.06	2.65 ± 0.04	2.94 ± 0.48
Acetate* (mmol L^−1^)	3.79 ± 0.68	3.28 ± 0.36	3.00 ± 1.33	2.34 ± 2.02	1.37 ± 0.49	0.27 ± 0.05	0.04 ± 0.10	0.05 ± 0.06	BDL^+^
Growth rate (h^−1^)	0.03 ± 0.03	0.01 ± 0.01	0.01 ± 0.01	0.02 ± 0.01	0.01 ± 0.01	0.02 ± 0.00	0.10 ± 0.01	0.18 ± 0.03	0.22 ± 0.05

* Concentration measured at the end of the reaction phase.

+ BDL, below detection limit.

Values are presented as average of the four cycles recorded and corresponding standard deviations.

Conversion-wise, at high light intensities (350 W m^−2^), acetate was fully depleted within the first hour of reaction (Figure 1B). At 264 and 175 W m^−2^, acetate reached a residual concentration of 0.05 ± 0.06 mmol L^−1^ and 0.04 ± 0.10 mmol L^−1^ at the end of the reaction phase, respectively (Table 1). At 87 W m^−2^, acetate was linearly decreasing over the 4 h of incubation, reaching a final concentration 0.27 ± 0.05 mmol L^−1^. At 30 W m^−2^, at the end of the incubation time, 2.34 ± 2.02 mmol L^−1^ of acetate was present at the end of the reaction phase. At the end of the reaction phase of the low light (15–0 W m^−2^) conditions, acetate was still present (3.10 ± 0.6 mmol L^−1^).

### Biomass growth rates followed a logistic distribution

**Figure 2:**
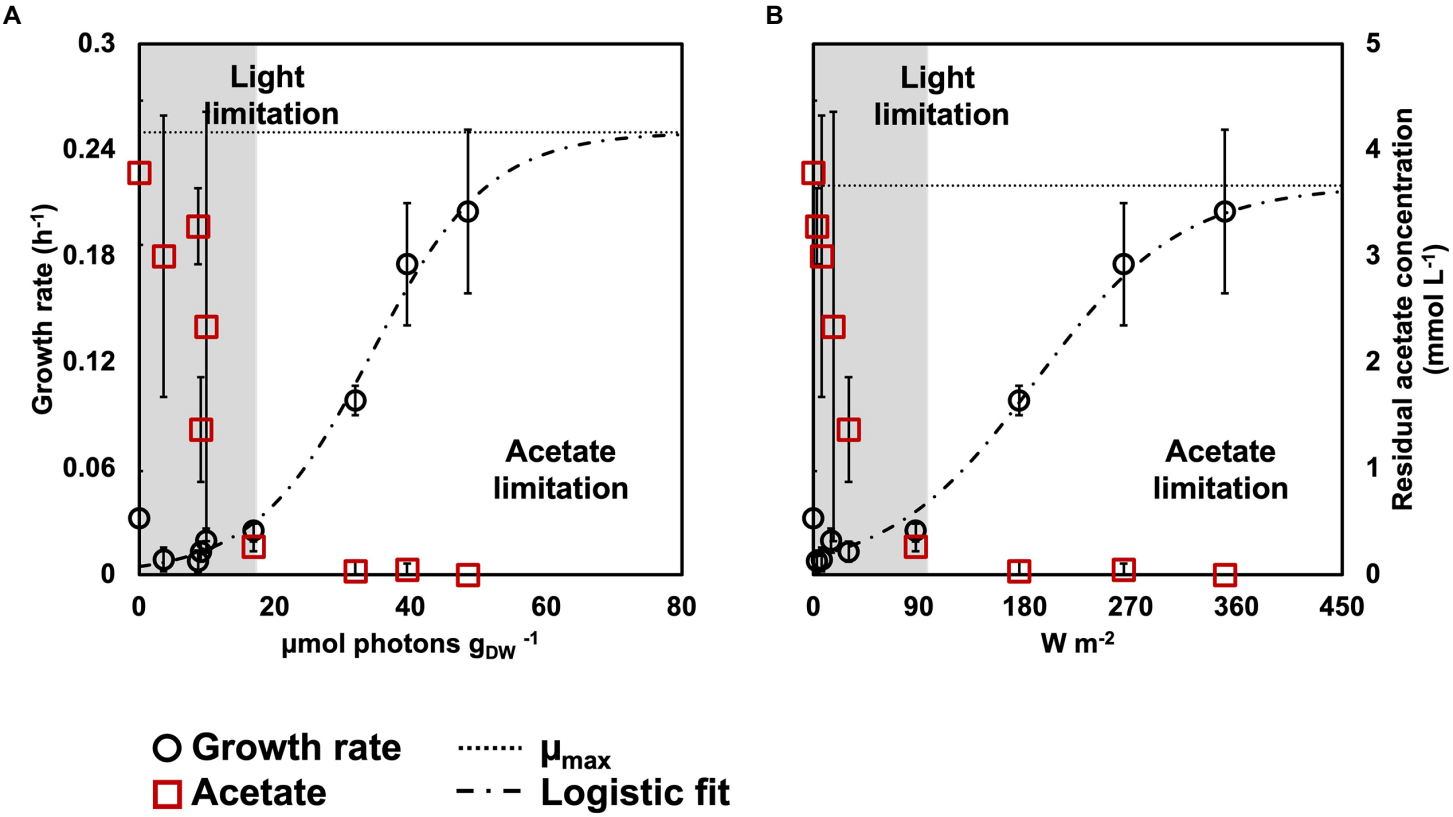
Biomass growth rates (black circles) and acetate concentration (red squares) in relation to rEX **(A)** and incident light intensity **(B)**. The growth rates fitted a logistic regression (dashed line), that allowed to identify the μ_max_ for the PPB enrichments (dotted line). Based on the acetate concentration at the end of the reaction phase, an acetate- and a light-limited condition were identified.

The growth rates decreased with the light intensity, from 0.22 ± 0.05 h^−1^ at 350 W m^−2^ to 0.03 ± 0.01 h^−1^ at 87 W m^−2^ to 0.008 ± 0.006 h^−1^ at 3 W m^−2^. The growth rates followed a logistic distribution, both considering the specific light supply rate (rEX; Figure 2A) and the incident light (Figure 2B). It was possible to identify two conditions, namely: (*i*) acetate-limited and (*ii*) light-limited conditions. The rEX delineated the two regimes. The cultures depleted in acetate at the end of the reaction phase were acetate-limited related to a rEX above 10 μmol_photons_ s^−1^ g_VSS_^1^. This rEX corresponded to an incident light of 87 W m^−2^. Below 10 μmol_photons_ s^−1^ g_VSS_^−1^, the cultures were light limited, and growth was reduced compared to the higher irradiances. The logistic curve (Eq. 5) fitted through the data allowed to identify three parameters: *a*, corresponding to the maximum growth rate (μ_max,_ h^−1^), *b*, a correction factor, and *c*, corresponding to a ‘half saturation constant’ for light. The factor *a* was identified with values of 0.24 and 0.22 h^−1^ retrieved from the growth rate curves in function of the rEX and the incident light, respectively. The factor *c* amounted to 34 μmol_photons_ s^−1^ g_VSS_^−1^ in the rEX-based fit and 189 W m^−2^ in the incident light-based fit. In Equation 5, *x* was defined as the light intensity (both as rEX and as incident irradiance, respectively), and *y* the specific growth rate.

### Community composition

**Figure 3:**
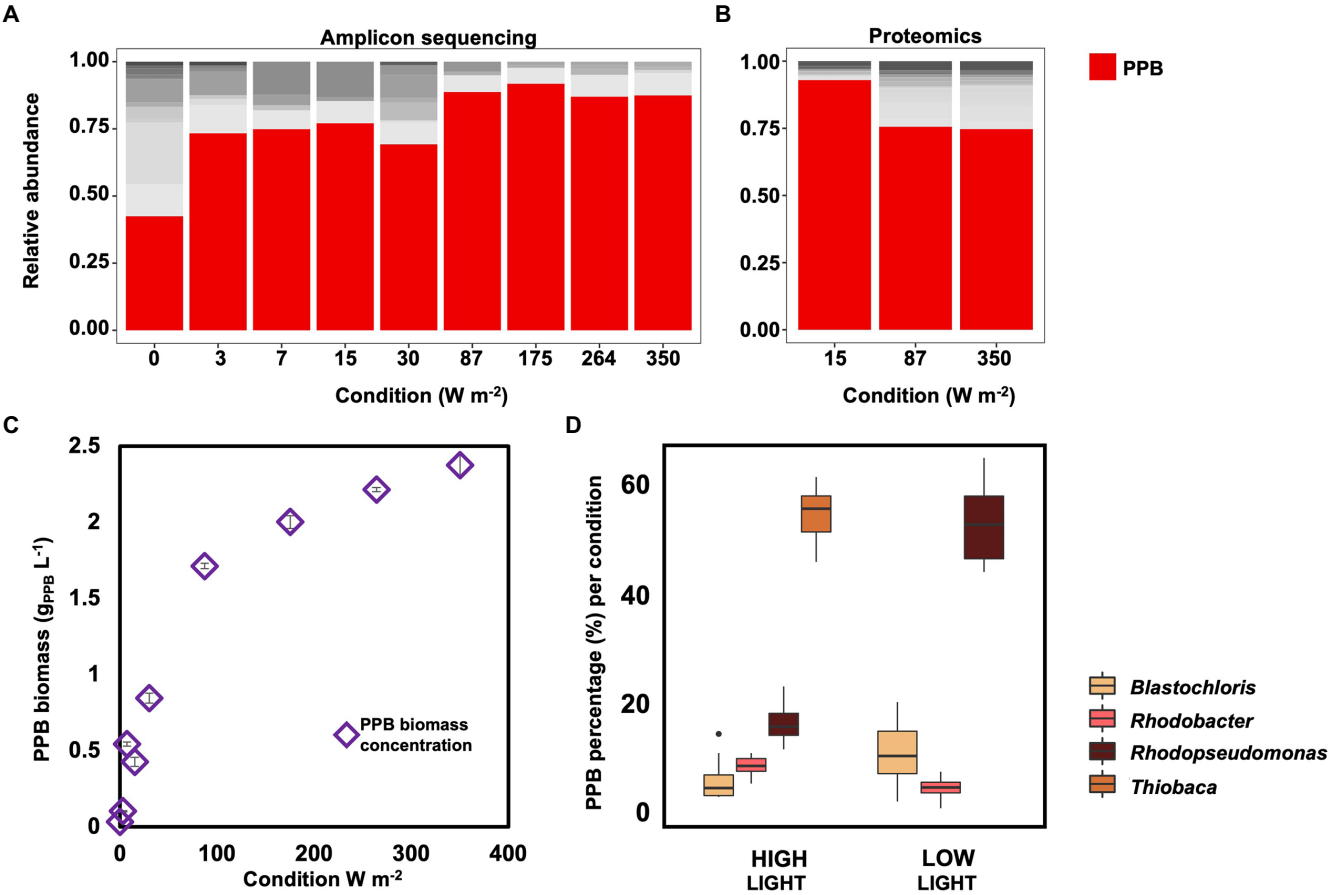
**(A)** Amplicon sequencing results for community composition. PPB genera (Blastochloris, Rhodobacter, Rhodopseudomonas and Thiobaca, red bars) were the dominant guild under all condition, accounting for ca 70% of the total community, excluding under dark condition. **(B)** Metaproteomic analysis of the community composition for selected samples. These results confirmed the amplicon sequencing results. **(C)** PPB concentration in the bulk liquid increased with the increase of incident light intensity. **(D)** Distribution of the main PPB genera based on amplicon sequencing results under high (350–87 W m^−2^) and low (30–0 W m^−2^) conditions. Notably, the genus Thiobaca was present only under high light conditions.

The taxonomic compositions of the bacterial communities obtained under the different irradiance treatments were measured by 16S rRNA gene amplicon sequencing. PPB were highly enriched, accounting for 81 ± 9% of the total reads under all irradiance conditions, except under dark conditions (i.e., 0 W m^−2^; Figure 3A). Under dark conditions, the PPB enrichment level decreased to 40%, while the genus *Dechloromonas* increased to 22%. Uncultured genera of the family of the *Rikenellaceae* accounted for 8%. The metaproteomic analysis confirmed the amplicon sequencing data. In the three samples analyzed by metaproteomics (350, 87, and 15 W m^−2^), more than 75% of the proteins were assigned to genera belonging to the PPB guild, namely *Rhodopseudomonas*, *Rhodobacter*, *Blastochloris*, and *Thiobaca* (Figure 3B).

The biomass concentration of PPB in the bulk liquid decreased with the decrease of the incident light, from 2.38 ± 0.07 g_VSS-PPB_ L^−1^ at 350 W m^−2^ to 0.03 ± 0.00 g_PPB_ L^−1^ under dark conditions (Figure 3C).

Among the PPB, *Thiobaca* was the dominant organism at high light intensities (350–87 W m^−2^), with a relative abundance of 56 ± 6%. *Rhodopseudomonas* and *Blastochloris* reached a relative abundance of 17 ± 4% of 7 ± 4%. Under low light conditions (30–3 W m^−2^) *Rhodopseudomonas* and *Blastochloris* were the dominant populations, with 54 ± 8% 12 ± 6%, respectively. The genus *Thiobaca* was not detectable under these conditions (Figure 3D). These differences might be attributed to the different inocula in the reactor under high and light irradiation, as explained later.

### Photopigment mass fraction

**Figure 4:**
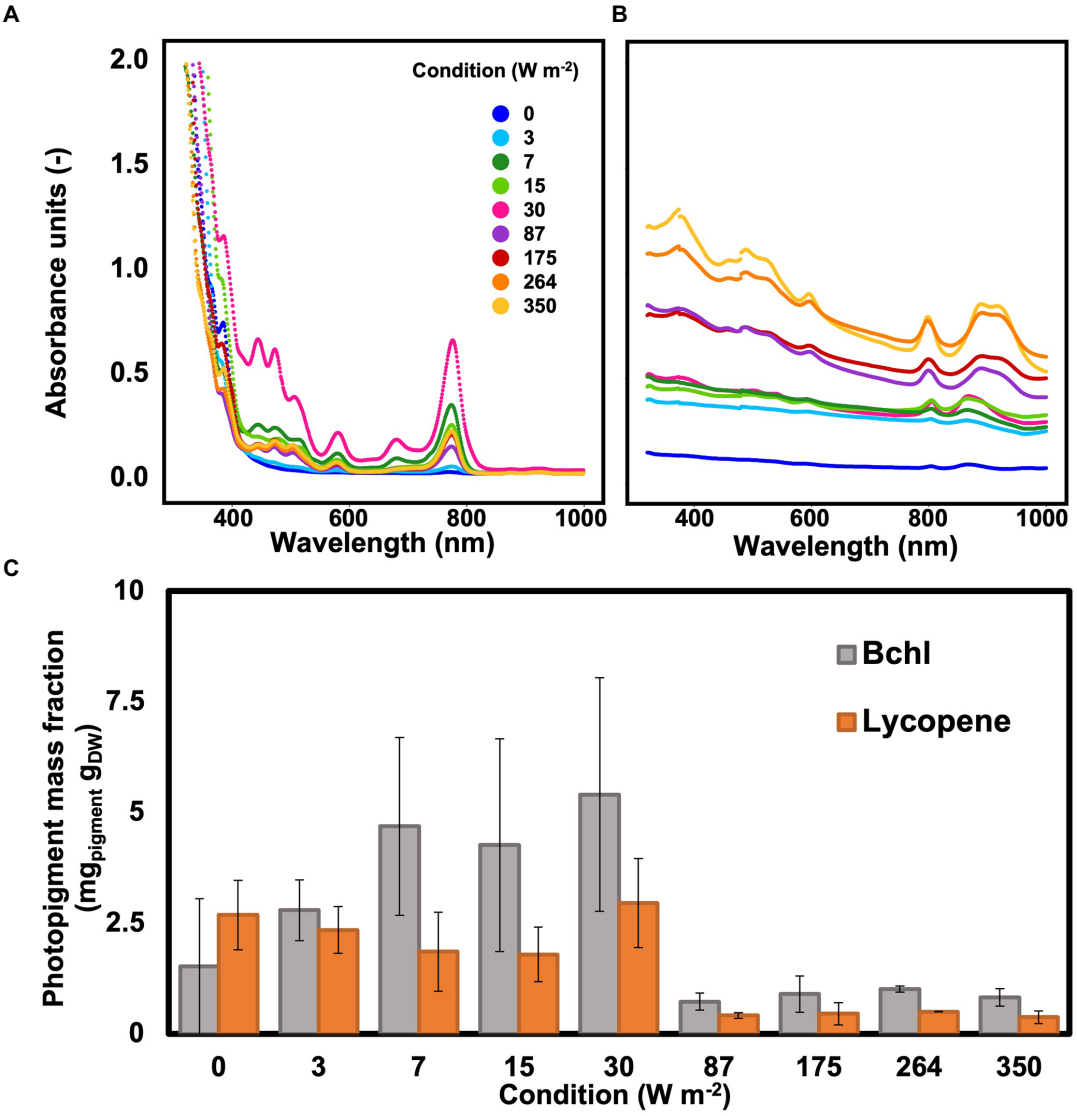
**(A)** Wavelength scan of PPB photopigments extract resuspended in hexane. The highest absorption peak was visible at 30 W m^−2^. **(B)** Wavelength scan of PPB whole cells. The absorption peaks in the bchl area shifted when changing high and low light intensities. **(C)**: The photopigments (bchl and lycopene) concentration in the cells was higher at lower light intensities compared to high light intensities.

Wavelength scans between 320 and 1,100 nm were recorded to determine the photopigment content in the whole cells of the biomass and in the pigments extract. Peaks in the wavelengths of the bacteriochlorophylls (800–900 nm) and carotenoids (between 400 and 500 nm) were detected under all conditions, including under dark, both in the extract and in the whole cells (Figures 4A,B). In the whole cells, under high light conditions (350–87 W m^−2^) two peaks were detected at 800 and 890 nm, corresponding to the bacteriochlorophylls (bchl). At low light intensities (30–0 W m^−2^), the bchl peaks were detectable at 805 and 866 nm. In the carotenoid area, peaks were detected at 322 and 370 nm under all conditions (Figure 4A).

In the extracts, the peaks for bchl were detected at 776 nm under all conditions. In the carotenoids area, peaks were present at 511, 475, 446, and 389 nm (Figure 4B). The bchl mass fraction at low light intensities (3.8 ± 1.7 mg g_VSS_^−1^ at 0–30 W m^−2^) was about 4.6 times higher than under high irradiance (0.8 ± 0.2 mg _VSS_^1^ at 87–350 W m^−2^). Similarly, the representative carotenoid (lycopene) showed a higher concentration at lower light intensities, with a maximum of about 3 mg g_VSS_^−1^ at 30 W m^−2^ (Figure 4C).

### Metaproteomics analysis

**Figure 5:**
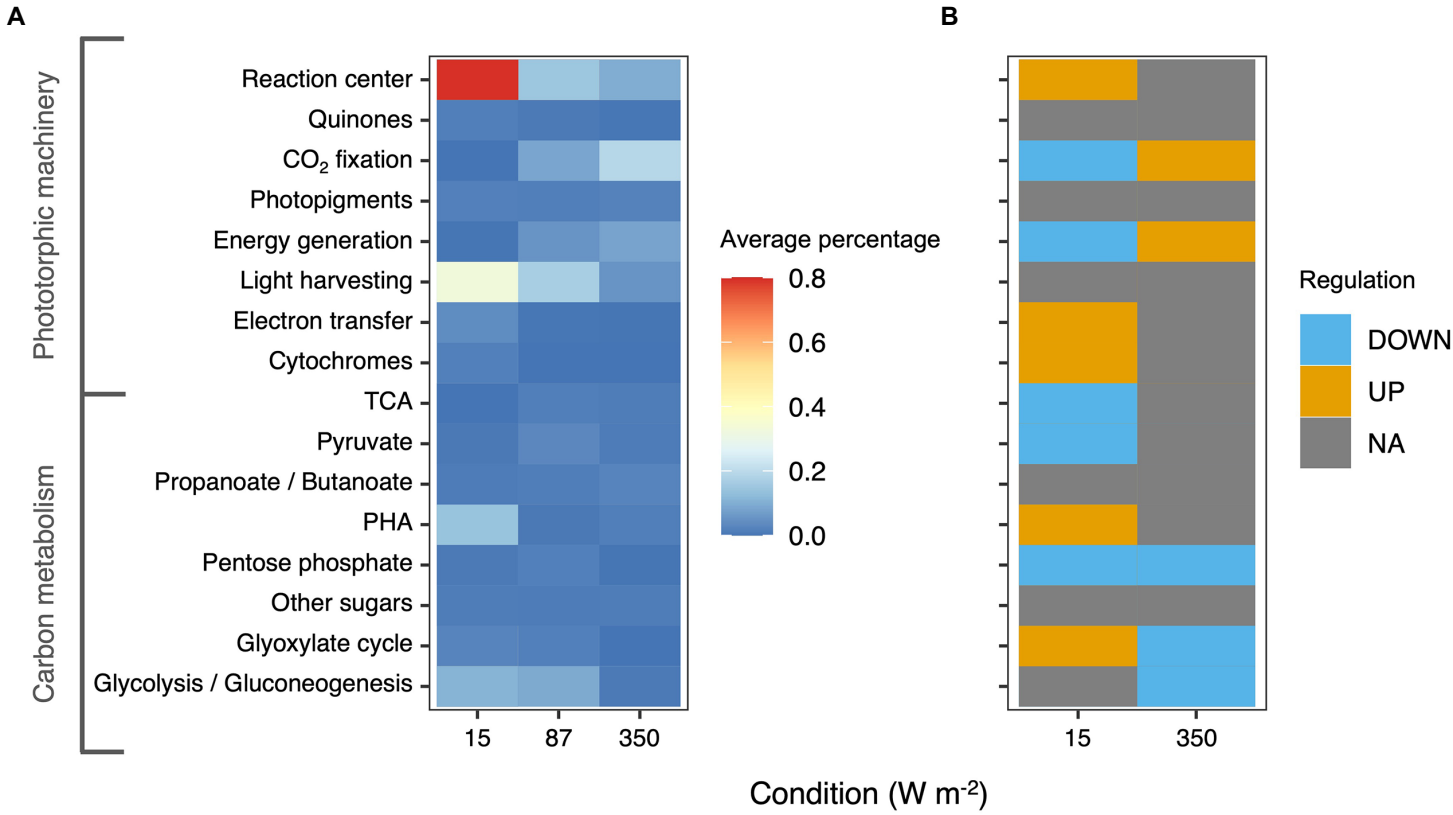
**(A)** Heatmap representing the average percentage of the protein expressed in each metabolic category based on KEGG classification. The heatmaps represents only carbon metabolism and phototrophic pathways. **(B)** Differential regulation of the different pathways under 15 and 350 W m^−2^ compared to the 87 W m^−2^ condition. Oxidative phosphorylation and photosynthesis pathways were upregulated in the 350 W m^−2^ condition and downregulated in the 15 W m^−2^ conditions compared to the 87 W m^−2^ condition.

Approximately 75% of the proteins identified at high confidence by metaproteomics belonged to the four main PPB genera that were also identified by amplicon sequencing, namely *Thiobaca*, *Rhodopseudomonas*, *Rhodobacter*, and *Blastochloris* (Figure 5).

Under all conditions, the most abundant proteins belonged to the phototropic machinery (reaction center proteins and cytochromes) and carbon metabolism. Following KEGG classification, the proteins for the phototrophic machinery accounted for 22.5 ± 0.7% of the total PPB proteins. The proteins of the reaction center (log_2_-foldchange = 0.97 ± 4.61) and the light-harvesting complexes (log_2_-foldchange = 3.58 ± 2.52) were significantly more expressed in the 15 W m^−2^ condition compared to the 87 W m^−2^ light limitation cutoff condition. In contrast with the results of the photopigment extraction, proteins involved in the photopigment formation were under-expressed at 15 W m^−2^ compared to the 87 W m^−2^ condition. At high light intensities (350 W m^−2^), proteins involved in phototrophic carbon fixation (log_2_-foldchange = 0.09 ± 1.88) and oxidative phosphorylation were more expressed compared to the 87 W m^−2^ condition.

In the carbon metabolism, at 15 W m^−2^ proteins involved in PHA metabolism were overexpressed compared to 87 W m^−2^ (log_2_-foldchange = 0.84 ± 4). Similarly, also the enzyme involved in the glyoxylate cycle, with a log_2_-foldchange of 0.61 ± 2.75 was overexpressed. At 350 W m^−2^, no significant difference was reported in the expression of the carbon metabolism enzymes compared to the 87 W m^−2^ condition. The complete metaproteomic profile of the analyzed samples is presented in Supplementary material 1.

## Discussion

### Photopigments content was inversely proportional to light intensity

For phototrophic organisms, energy is derived by light. Based on the irradiation conditions, PPB adapt the number and disposition of photopigments in their membranes to maximize the light harvest (Gardiner et al., 1993; Brotosudarmo et al., 2011). In line with previous observations (Liu et al., 2019), we report an increase in photopigment content (both bacteriochlorophylls and carotenoids) with a decrease of light intensity. In fact, bacteriochlorophyll and carotenoid mass fractions were, respectively, 4.6 and 10 times higher under low light conditions compared to high light conditions. The variation in the photopigment content of the biomass can be attributed to different factors. The decrease of photopigments is an acclimatization strategy to high light intensities. Muzziotti et al. (2016) have reported that *Rhodopseudomonas* increases 6-fold its bacteriochlorophyll mass fraction and 2.5-fold its carotenoids content when light intensity was switched from 250 to 1,500 μmol_phtotons_ m^−2^ s^−1^ (corresponding to *ca.* 33 and 190 W m^−2^) under anaerobic conditions. By reducing the antenna size and consequently the amount of light absorbed, PPB protect the cells from photodamage induced by high light intensities (Muzziotti et al., 2017; Bayon-Vicente et al., 2020). Furthermore, phototrophic growth is linked to the photopigment content and activation state of the reaction centers that harvest light and convert it into chemical energy (Saikin et al., 2014). To compensate for the lower energy supply, PPB increased the mass fraction of photopigments.

The variation in photopigment mass fraction might also be linked to the taxonomic composition of the enrichment. The microbial community was different in the two experimental set-ups (high and low light intensities, Figure 3D). At low light intensities, the community was enriched for the genus *Rhodopseudomonas*. At high light intensities, instead, the most abundant genus was *Thiobaca*. Each organism has a different distribution of the photopigment (Bullough et al., 2009; Timpmann et al., 2014). To maximize the efficiency of energy capture, PPB combine protein and pigments in the light-harvesting complexes and reaction center (Fixen et al., 2016). The differences in protein binding of the pigments influence the stability of the complexes (Mizoguchi et al., 2012), and potentially have affected the extraction yields in our enrichments. However, the taxonomic difference between *Thiobaca* and *Rhodopseudomonas* cannot be interpreted as a selection mechanism caused by light irradiance, but as an inoculum effect such as explained in the next section.

### PPB were enriched under all conditions

PPB formed the dominant guild under all conditions, confirming the possibility to efficiently enrich them on acetate as electron donor and carbon source and with IR light as energy source (Alloul et al., 2019; Cerruti et al., 2020b). The supply of IR light selectively enriches for PPB and prevents the growth of other phototrophs, such as green/blue phototrophic organisms that extract their energy on other wavelengths. This prevents formation of oxygen and ensures all organic matter is available for the PPB. The PPB enrichment was confirmed both at genetic (amplicon sequencing) and functional levels (metaproteomics).

The taxonomic variation from a *Thiobaca*- to a *Rhodopseudomonas*-dominant community was due to the inoculum rather than to a light effect. The low light conditions were applied to the same parent reactor 4 months after the high light conditions. We previously reported the predominance of the genus *Rhodopseudomonas* at high light intensities (> 300 W m^−2^) under equal SBR cultivation conditions as applied here (Cerruti et al., 2020b). In a batch system, the selective pressure is determined by the growth rates (Winkler et al., 2017). No information is currently available for the growth kinetics of *Thiobaca*. Pure cultures of *Rhodopseudomonas* present higher growth rates (μ_max_ = 0.15 h^−1^ (Cerruti et al., 2020a)) compared to other PPB pure cultures like *Rhodobacter* or *Blastochloris* under acetate feed (0.10 and ca 0.03 h^−1^ respectively) and was therefore predominant under SBR conditions (LaClair, 2006; Zavala et al., 2019). We were not able to detect the cause of the presence of *Thiobaca* in the initial mother culture. Potentially, competition between purple sulfur bacteria (*Thiobaca*) and purple non-sulfur bacteria (*Rhodopseudomonas*, *Rhodobacter*, and *Blastochloris*) might be linked to the accumulation of sulfur compounds in the medium (not measured in this study). Competition phenomena between PNSB and PSB should be further researched in the future.

The PPB enrichment was stable under all IR light intensities, with a relative abundance above 75%, except for the dark condition (0 W m^−2^). In presence of IR light, PPB show relatively high growth rates, ranging between 0.03 and 0.3 h^−1^ (Alloul et al., 2019). Few organisms can grow under anaerobic conditions with acetate as sole carbon and electron source, since acetate is already a fermentation product. Acetoclastic organisms, like the slow-growing methanogenic archaea, present a μ_max_ of 0.004–0.013 h^−1^ (Kotsyurbenko et al., 2019). In the reactor, the SRT was controlled at 31 h, implying that the minimum growth rate to be retained in the bulk liquid was set to 0.03 h^−1^. The taxonomic analyses showed that no methanogens were detected both at 16S rRNA gene level and at protein level. Under dark anaerobic conditions, PPB cannot grow on acetate without external electron acceptors (Yen and Marrs, 1977), which were not present in the cultivation medium. Possibly, the high relative abundance of PPB under dark conditions can be attributed to the biomass retention during the settling phase. The chemotrophic growth of *Dechloromonas* (present at 22% under dark conditions) explains the apparent higher growth rates measured for the biomass under dark condition compared to other low light intensities. Under low-light conditions, the growth rates were almost equal to the SRT (0.03 h^−1^). Iasimone et al. (2018) have reported an increase in the length of the lag phase with the decrease of light intensity. Possibly, at low light intensities, the PPB enrichment was still in the lag phase. An increase of the reaction length might lead to a biomass increase, and consequently higher carbon uptake and photopigment recovery.

### Enzymatic expression changed with light intensity

In the proteomic analysis, it emerged that at low light conditions the proteins responsible for the light harvest were overexpressed compared to higher light intensities. These results match with the hypothesis that, with lower irradiances, cells require to produce more light-harvesting complexes to be able to sustain the energy requirements. Interestingly, the proteins for the formation of accessory pigments were expressed under all conditions at the same level.

Enzymes involved in the electron transport chain and ATP generation were overexpressed at high light intensities, similarly to what has been reported for microalgae (Toyoshima et al., 2019). The higher ATP production rate can have enhanced the carbon fixation (photosynthesis) pathway, whose enzymes were overexpressed at high light conditions (350 W m^−2^). In fact, the Calvin-Benson-Bassham cycle is a known mechanism of electron reallocation (McKinlay and Harwood, 2010). The energy-expensive CO_2_ fixation regulates the redox balance by increasing the fixation rates when an excess of ATP is produced through the electron transport chain (Alsiyabi et al., 2019). Polyhydroxyalkanoates (PHA) formation in PPB is usually linked to redox stress conditions (Bayon-Vicente et al., 2020; Cerruti et al., 2020a). The enzymes linked to the PHA granules were upregulated in the low light condition (15 W m^−2^), namely proteins belonging to the phasin family. However, enzymes for PHA formation were not detected. The function of the phasin proteins under this condition is unclear.

The high standard deviations reported for the log2-foldchange can be attributed to the microbial community composition. For example, at low light intensities, where *Rhodopseudomonas* was the most abundant genus, 284 proteins belonging to *Rhodopseudomonas* were over-expressed compared to the 87 W m^−2^ condition, 58 proteins were not significantly differently expressed, and only 17 proteins were downregulated. The opposite was reported for *Thiobaca* (the most abundant genus at high light intensities): only 2 of its proteins were upregulated at low light conditions, whereas 268 were downregulated, and 32 were not significantly differently expressed.

The over-expression of the glyoxylate cycle enzymes in the 15 W m^−2^ condition can also be linked to the community composition. Carbon metabolism in PPB is species-specific, i.e., the different organisms use different pathways to catabolize acetate. For instance, the genus *Rhodopseudomonas* utilizes the glyoxylate shunt to metabolize the acetate (Albers and Gottschalk, 1976), whereas *Rhodobacter* uses the EMC pathway (Alber et al., 2006). An enrichment of *Rhodopseudomonas* in the reactor can explain an enrichment of the glyoxylate cycle proteins at low light intensities.

### Growth rates are dependent to light availability

Numerous kinetics models have been constructed to correlate the growth rates of phototrophic organisms to light intensity and substrate availability (Lee et al., 2015). Light and nutrients are complementary factors contributing to growth kinetics of phototrophs. Under light-saturating conditions, the concentration of the limiting substrate (electron donor or C-P-N nutrients) defines the growth rates, as described by the Monod model (Doran, 2013). Under nutrient-saturating conditions, growth rates are instead controlled by light intensity. At light saturation level, the maximum growth rate is achieved (Undurraga et al., 2016). Above this point, growth is inhibited (photoinhibition). Below the saturation level, growth is light-limited. At present, the definition of a univocal kinetic model for phototrophic growth is not possible, as numerous factors influence the growth rates (Lee et al., 2015). Furthermore, the kinetic parameters to define saturation and inhibition constants are species-specific (Huesemann et al., 2013), further complicating the task.

Two growth regimes were delineated for the PPB biomass, namely light-limited and acetate-limited growth. At high light intensities (350–175 W m^−2^), the complete depletion of the carbon source and e-donor (acetate) switched off the biomass growth, indicating that acetate was the limiting compound. Under these conditions, the biomass grew with a growth rate close to the calculated μ_max_ (0.10–0.22 h^−1^). At low light intensities, the biomass displayed growth rates almost 8 times lower than under high irradiance. The presence of residual acetate at the end of the SBR reaction phase suggested that the culture was light-limited. The biomass growth rates were approximated by a logistic expression. The logistic function has been used for more than one century in ecology to describe the growth of populations (Verhulst, 1838; Kendrick and Kesava Pai, 1911). More recently, it has been used to describe the growth of some PPB (Koku et al., 2003; Erogˇlu et al., 2004, 2008). In the logistic model, the growth of a population is described in terms of intrinsic rate of increase, or μ_max_ (maximum growth rate), and the carrying capacity (maximum population size at the available resource). In our study, the fitted μ_max_ was 0.22–0.25 h^−1^, in the range of the reported μ_max_ for PPB (Hunter et al., 2009). The carrying capacity was defined at a rEX of 10 μmol_photons_ s^−1^ g_VSS_^−1^, or an incident light of 87 W m^−2^. These values can be considered as the ‘half saturation constant’ for light (K_L_). The K_L_ of the rEX is particularly important for process design, as it allows to determine the limit for biomass growth. Our result suggests that light is governing all the other metabolic processes, by providing energy to the cells, and an ATP balance would be required to further sustain this hypothesis. ATP production and consumption in PPB have been described in metabolic models (Golomysova et al., 2010), but an accurate measurement of the ATP produced by PPB per photon absorbed has not yet been reported.

### Light intensity impacted PPB systems

In phototrophic systems, light provides energy to cellular growth. To design a photobioprocess, it is necessary to accurately manage the light attenuation, as it impacts the reactor performances. The Lambert–Beer equation describes the light attenuation due to biomass concentration and photopigment content, but it is lacking the refraction and scattering component. On the other hand, the specific light supply rate describes how much light each gram of cells can experience but does not consider the optical effects. Numerous models have been developed to link nutrients and light availability to the biomass kinetic parameter [reviewed by Lee et al. (2015)]. These models are based on pure culture studies, and do not consider the interactions, as competition, mutualism, or synthrophy, between organisms in a community.

In the PPB enrichment, we found that the specific growth rates were correlated to the light intensity available for the cells through a logistic regression. The data fitting provided insights the kinetic parameters of the PPB culture, as the limit for photoinhibition phenomena (μ_max_ = 0.25 h^−1^ at 80 μmol_photons_ s^−1^ g_VSS_^−1^) and the half saturation constant for light (K_L_ = 10 μmol_photons_ s^−1^ g_VSS_^−1^). Growth kinetic models are needed to understand PPB growth and therefore to optimize the cultivation condition. For scale-up purposes, mathematical modeling proves to be a useful tool to predict the microbial behavior. Future research should address a multivariate predictive analysis of the kinetic parameters of PPB enrichments.

PPB form a complex microbial guild, whose metabolism is primarily photoorganoheterotrophic. By changing the incident light, we elucidated the mechanisms of adaptation of a PPB community to variation in energy supply. Our findings contribute to paving the way for PPB-based bioprocesses.

We can conclude that:

- The PPB guild was enriched above 70% of the total community under all conditions in presence of IR light. In the dark, PPB were still dominant under the experimental period, but only at 40%, getting in competition with *Dechloromonas* which can anaerobically respire acetate with sulfate.
- PPB growth can be either nutrient-limited or light-limited. At a flux rate (rEX) above 10 μmol_photons_ s^−1^ g_VSS_^−1^, the enrichment was acetate-limited. Below this point it was light limited. Notably, this value is important for process design.
- PPB growth rates fitted a logistic function. This allowed to identify the maximum growth rate (μ_max_ = 0.25 h^−1^) and the half saturation constant for light (K_L_ = μmol_photons_ s^−1^ g_VSS_^−1^) of the PPB biomass.
- Photopigment mass fraction was *ca.* 5 times higher at low light intensities compared to high light intensities. This can be due both to physiological responses and to changes in community composition.
- PPB adapted their enzymatic expression to respond to the light stress. At low light intensities the proteins involved in light-harvesting processes were more expressed, whereas at high light intensities PPB activated mechanisms involved in stress response.

## Data availability statement

The datasets presented in this study can be found in online repositories. The names of the repository/repositories and accession number(s) can be found in the article/Supplementary material.

## Author contributions

MC: conceptualization, experimental research, data curation, and writing. J-HK: experimental research. MP: proteomic analysis. ML: formal analysis, review, and supervision. DW: review and editing, supervision, and funding acquisition. All authors contributed to the article and approved the submitted version

## Funding

This study was financed by the tenure-track start-up grant of the Department of Biotechnology of the Faculty of Applied Sciences of the TU Delft (DW, PI).

## Conflict of interest

The authors declare that the research was conducted in the absence of any commercial or financial relationships that could be construed as a potential conflict of interest.

## Publisher’s note

All claims expressed in this article are solely those of the authors and do not necessarily represent those of their affiliated organizations, or those of the publisher, the editors and the reviewers. Any product that may be evaluated in this article, or claim that may be made by its manufacturer, is not guaranteed or endorsed by the publisher.

## Footnotes

^1^ www.mathworks.com
