# Supplementary figures and images for: Light intensity defines growth and photopigment content of a mixed culture of purple phototrophic bacteria
Source: Front Microbiol. 2022 Oct 19;13:1014695. doi: 10.3389/fmicb.2022.1014695 (PMC9628752; doi:10.3389/fmicb.2022.1014695)

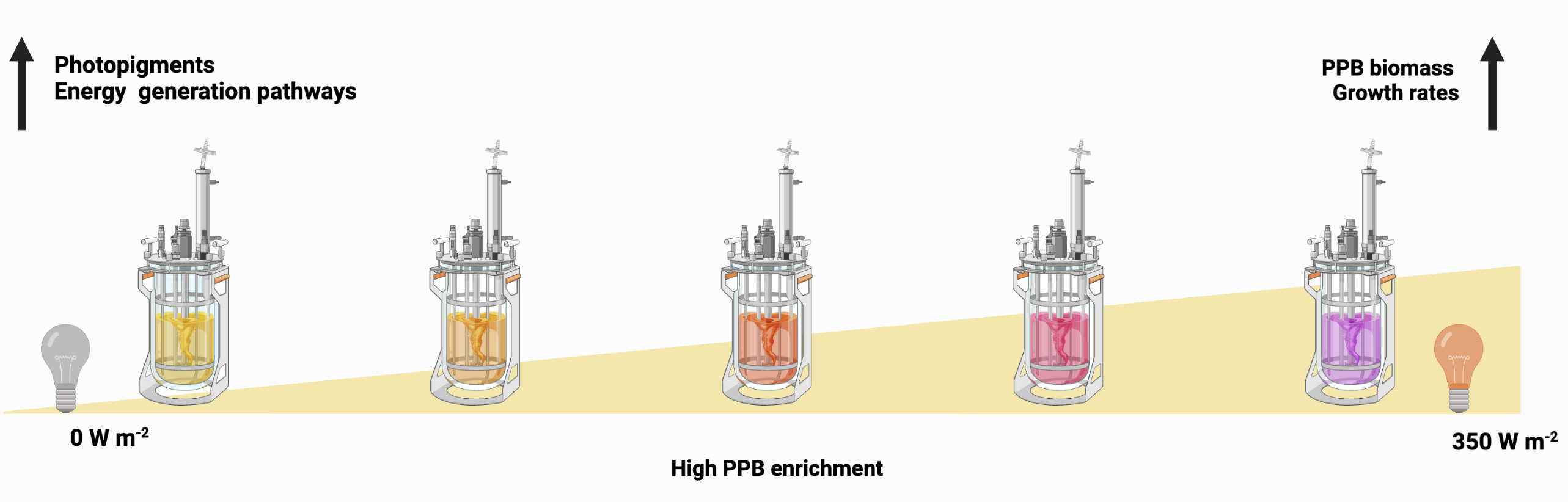

Supplement: Supplementary file 1 [file Image_1.JPEG]
